# AST3DRNet: Attention-Based Spatio-Temporal 3D Residual Neural Networks for Traffic Congestion Prediction

**DOI:** 10.3390/s24041261

**Published:** 2024-02-16

**Authors:** Lecheng Li, Fei Dai, Bi Huang, Shuai Wang, Wanchun Dou, Xiaodong Fu

**Affiliations:** 1 School of Big Data and Intelligent Engineering, Southwest Forestry University, Kunming 650224, China; lecheng@swfu.edu.cn (L.L.); bihuang@swfu.edu.cn (B.H.); shuaiwang@swfu.edu.cn (S.W.); 2 State Key Laboratory for Novel Software Technology, Department of Computer Science and Technology, Nanjing University, Nanjing 210008, China; douwc@nju.edu.cn; 3 Faculty of Information Engineering and Automation, Kunming University of Science and Technology, Kunming 650500, China; xiaodong_fu@hotmail.com

**Keywords:** traffic congestion prediction, 3D convolution, 3D residual unit, self-attention mechanism, spatio-temporal attention

## Abstract

Traffic congestion prediction has become an indispensable component of an intelligent transport system. However, one limitation of the existing methods is that they treat the effects of spatio-temporal correlations on traffic prediction as invariable during modeling spatio-temporal features, which results in inadequate modeling. In this paper, we propose an attention-based spatio-temporal 3D residual neural network, named AST3DRNet, to directly forecast the congestion levels of road networks in a city. AST3DRNet combines a 3D residual network and a self-attention mechanism together to efficiently model the spatial and temporal information of traffic congestion data. Specifically, by stacking 3D residual units and 3D convolution, we proposed a 3D convolution module that can simultaneously capture various spatio-temporal correlations. Furthermore, a novel spatio-temporal attention module is proposed to explicitly model the different contributions of spatio-temporal correlations in both spatial and temporal dimensions through the self-attention mechanism. Extensive experiments are conducted on a real-world traffic congestion dataset in Kunming, and the results demonstrate that AST3DRNet outperforms the baselines in short-term (5/10/15 min) traffic congestion predictions with an average accuracy improvement of 59.05%, 64.69%, and 48.22%, respectively.

## 1. Introduction

With the growing number of vehicles in cities, traffic congestion has increased drastically [1]. Urban traffic congestion can lead to a variety of problems, such as environmental pollution [2], increased commute times [1], and an increase in the frequency of road accidents [3]. Currently, with the rapid development of intelligent transport systems (ITS) [2], traffic congestion prediction has become an indispensable component, which can greatly improve the effectiveness and capacity of road networks. For example, if commuters can predict congestion information in advance, the high congestion problem can be avoided by switching routes.

Compared to traditional traffic flow [1,3,4,5] prediction and traffic speed prediction [6,7,8,9], urban traffic congestion prediction mainly focuses on congestion levels of road networks in cities. However, forecasting congestion levels of road networks is very challenging due to the following two complex factors:

- Spatio-temporal correlation. Traffic data show correlations both in space and time [10]. On the one hand, the observations of different road segments are correlated with each other through road connectivity. This may lead to nearby correlations and distant correlations in space. On the other hand, traffic data show closeness, periodicity, and a trend in time [10].
- Spatio-temporal heterogeneity. The extent of spatial and temporal correlations of traffic data is not constant in the spatial and temporal dimensions. Influenced by the functional areas of the city and the occurrence of emergencies, traffic congestion presents the characteristics of regional concentration and multi-point occurrence in space and the characteristics of random occurrence in time. The characteristics of spatial and temporal heterogeneity are obvious.

Extensive research has been carried out to accurately predict traffic conditions. These methods can be roughly divided into three types: statistical analysis methods, machine learning methods, and deep learning methods. Statistical analysis methods, such as historical average algorithms (HA) [11] and autoregressive integrated moving average models (ARIMA) [12], use ideal parameter models for traffic prediction by capturing the linear features of traffic data. However, they are unsuitable for modeling nonlinear features, leading to low prediction accuracy. Machine learning methods include support vector machines (SVM) [13], support vector regression (SVR) [14], and other machine learning methods. Although these methods can use shallow models to learn the low-dimensional nonlinear features of traffic data, they may struggle to provide accurate predictions when working with highly complex and changeable traffic data. Deep learning methods, such as STResNet [10], ST-3DNet [15], and STGCN [16], rely on convolutional neural networks (CNNs) [17], recurrent neural networks (RNNs) [18], or graph convolutional networks (GCNs) [19] to learn the complex dynamic high-dimensional nonlinear features of traffic data. However, these existing methods still have the problem of the inadequate modeling of spatio-temporal features, especially spatio-temporal heterogeneity.

Fortunately, the self-attention mechanism [20,21,22,23] can effectively model the spatio-temporal heterogeneity of traffic data. Inspired by this, we propose an attention-based spatio-temporal 3D residual neural network, named AST3DRNet, to forecast the congestion levels of road networks in a city. It aims to model both spatio-temporal correlations and spatio-temporal heterogeneity. Specifically, we propose a 3DCon (3D convolutions) module to capture various spatio-temporal correlations and an STA (spatio-temporal attention) module to model dynamic spatio-temporal heterogeneity.

The main contributions of this paper are summarized as follows:

- AST3DRNet designs a novel 3DCon module to simultaneously model spatio-temporal correlations of traffic congestion data. Compared to 2D convolutions, the 3DCon module can better model the temporal information of traffic congestion data using 3D convolutions.
- AST3DRNet designs a novel STA module to explicitly model the spatio-temporal heterogeneity of traffic congestion data. The STA module employs a self-attention mechanism to emphasize meaningful features and suppress unnecessary ones along the spatial and temporal dimensions.
- We evaluated AST3DRNet on the real-world traffic congestion dataset. The experimental results demonstrate that our AST3DRNet outperforms four baseline models.

## 2. Related Work

Traffic prediction approaches can be classified into three major categories: statistical analysis methods, machine learning methods, and deep learning methods [15].

**Statistical analysis methods**. The historical average (HA) [11] algorithm uses historical average data for traffic prediction. This method calculates the average historical traffic volume of a particular time interval (such as an hour, day, week, month, etc.) and uses this average as the predicted value. The main advantage of HA is its simplicity and low computational cost. The ARIMA [12] method can be used to capture the trend, seasonality, and autocorrelation of traffic data. Although ARIMA requires certain assumptions, such as data stationarity and constant variance, it is still widely used in traffic prediction because of its interpretability. In addition, vector autoregression (VAR) [10] and the vector error correction model (VECM) [24] are commonly used for traffic forecasting. The VAR method is a multivariate time-series model that can capture the interdependencies between different road sections. The VECM model is an extension of the VAR model that introduces relative error to correct the accumulation of errors, making results more reliable and accurate. These methods can provide sophisticated and interpretable prediction models, but they require rigorous data preprocessing and present difficulties when choosing the model hyperparameters.

**Machine learning methods**. The SVR [14] can capture low-dimension nonlinear relationships between traffic variables. The enhanced K-nearest neighbor (K-NN) [25] is a short-term traffic prediction method that suppresses regions of weak correlation by the weighted enhancement of neighboring regions. The graph processing-based traffic estimation (GPTE) [26] models a road network structure as graph-structured data for traffic estimation; it can learn the low-dimensional nonlinear features from traffic data. A gradient-boosted regression tree (GBRT) [27] is also a popular short-term traffic prediction algorithm, which can predict traffic speeds based on weather conditions, historical traffic data, and road networks. In addition, the extreme learning machine (ELM) [28,29,30] is also a typical method that can be applied to traffic forecasting. Chai et al. [31] proposed the GA-KELM to forecast traffic flow. This method reduces the overfitting problem and achieves more accurate prediction performance by optimizing the kernel limit-learning machine with a genetic algorithm. However, these methods are mainly suitable for simple short-term traffic forecasting and lack the ability to model complex long-term traffic patterns.

**Deep learning methods**. Generally, CNNs use a stack of convolutional operations to extract the spatial information of traffic data. For example, Zhang et al. [10] proposed an ST-ResNet for traffic flow prediction, which employs three residual components and fusion components to model spatial–temporal correlations. Dai et al. [11] arranged an ST-InNet for traffic flow prediction, which leverages two Inception networks to capture correlation information. Guo et al. [15] proposed an ST-3DNet based on ST-ResNet, which combines 3D convolution and traditional residual units for modeling the spatio-temporal dependencies and spatial heterogeneity. These convolutional neural network models can effectively construct spatio-temporal correlations, but they are ambiguous in explaining spatio-temporal heterogeneity.

RNNs are matched for modeling temporal information. For example, Long-Short-Term Memory (LSTM) [18] is used to extract traffic time series dependency but suffers from the gradient vanishing problem. Seq2Seq [32] can predicate future traffic flow conditions based on historical traffic flow data by modeling temporal dependencies, where the input is two different sequences of matrices. Li et al. [33] presented a DCRNN for traffic graph data prediction, which utilizes two different computational mechanisms to capture spatial and temporal dependencies, respectively. This recurrent model has a greater short-term memory capacity and can effectively process short-term temporal information, but its ability to model spatial features and long-term dependencies is deficient.

In comparison to CNN- or RNN-based methods, GCNs are capable of modeling graph-structured (i.e., non-Euclidean) traffic data. For example, Yu et al. [16] proposed an STGCN, which relies on graph convolutions and gated temporal convolutions to model traffic feature information. Song et al. [34] proposed an STSGCN, which builds the synchronization modeling mechanism to model spatial–temporal correlations. Bai et al. [35] proposed an AGCRN, which mainly models traffic dependencies through node adaptive learning and adaptive graph generation. Choi et al. [36] proposed an STG-NCDE, which designs two neural-controlled differential equations for modeling traffic correlations. Prediction methods based on graph convolution are greatly limited by the number of nodes and are prone to overfitting, making model training difficult.

In addition, some researchers introduce attention mechanisms [20,21] into the traffic prediction domain. Guo et al. [37] proposed a spatio-temporal graph convolutional neural network (ASTGCN) with the attention mechanism, which combines spatio-temporal attention with a spatio-temporal graph convolution neural network and can efficiently model dynamic spatio-temporal dependence. Xu et al. [38] proposed a spatio-temporal transformer network (STTN) for traffic flow forecasting; this method is also a hybrid model that combines a transformer structure with a graph convolutional neural network. Jiang et al. [39] considered that the propagation of traffic states is delayed and therefore constructed a propagation delay-aware dynamic long-range transformer (PDFormer) model to predict the traffic flow. This method uses a multi-head attention mechanism and each head extracts different spatio-temporal features to achieve better prediction results. Ramana et al. [40] combined the CNN, vision transformer, and LSTM to construct a prediction model, which achieved better prediction through the stage-by-stage extraction of spatio-temporal features.

Different from all these methods, AST3DRNet can model both spatial and temporal correlation, while spatial heterogeneity and temporal heterogeneity are also taken into account. In addition, AST3DRNet introduces residual structure to improve the convergence speed of the model and enhance the optimization efficiency.

## 3. Problem Definition

**Figure 1:**
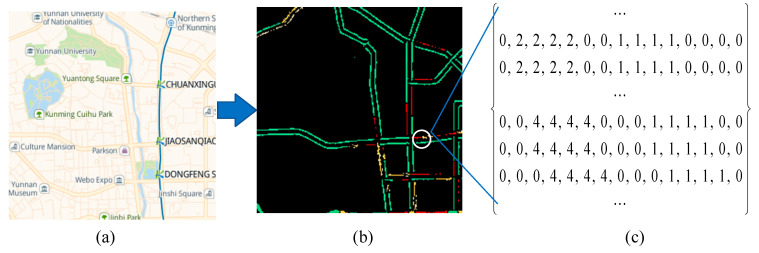
An illustration of encoding a traffic congestion image into a traffic congestion matrix. (**a**) A sample of a traffic congestion image captured from the Baidu Map. (**b**) A sample of the resulting image with only road networks and background. (**c**) A sample of a traffic congestion matrix.

Figure 1a shows a raw traffic congestion image captured from the Baidu Map, which provides city-wide and real-time congestion levels of road networks. It has four levels, namely green, yellow, red, and severe red, where green denotes traffic-free, yellow denotes traffic slow, red denotes traffic jam, and severe red denotes severe traffic jam, respectively.

To encode raw traffic congestion data, we require two steps to convert a raw traffic congestion image into a traffic congestion matrix.

First, we define the pixel congestion level (PCL). A traffic congestion image consists of a collection of pixels, where the value of each pixel is represented in RGB format. Thus, each pixel’s PCL can be measured by the upper and lower boundary values for red color, green color, and blue color, respectively. In short, PCL can be defined as follows:

(1) $$\mathrm{PCL}=\left\{\begin{array}{l} \mathrm{green},\;\;\;\;\;\;\;\;\;\;\;\;\;127<R<200,\;211<G<230,\;81<B<180\\ \mathrm{yellow},\;\;\;\;\;\;\;\;\;\;\;\;0<R<18,\;0<G<18,\;181<B<199\\ \mathrm{red},\;\;\;\;\;\;\;\;\;\;\;\;\;\;\;\;\;\;72<R<94,\;211<G<233,\;250<B<272\\ \mathrm{severe}\;\mathrm{red},\;\;\;\;14<R<36,\;14<G<36,\;232<B<254\\ \mathrm{black},\;\;\;\;\;\;\;\;\;\;\;\;\;\;\;R=0,\;G=0,\;B=0 \end{array}\right.$$

where the green, yellow, red, and severe red indicate smooth, slow, congested, and severely congested traffic conditions, respectively. In addition, the black denotes the background of a traffic congestion image.

Figure 1b shows the resulting image, with only road networks and background, from the raw traffic congestion image shown in Figure 1a through image mask and bitwise operation.

Second, we turn the resulting image with only road networks and a background into a traffic congestion matrix. In the traffic congestion matrix, shown in Figure 1c, the range of values for each element is 0 to 4. These values denote the background color, the green color, the yellow color, the red color, and the severe red color, respectively.

*Problem 1 (Traffic Congestion Prediction)*: Given historical traffic congestion matrices $\{X^{t-n+1},X^{t-n+2},\ldots ,X^{t}\}\in {\mathbb{R}}^{H\times W}$, where (*H*, *W*) is the resolution of the matrices, traffic congestion prediction can be formulated as predicting the future congestion matrices over *T* time steps, which is defined as follows:

(2) $$\left(X^{t+1},X^{t+2},\ldots ,X^{t+T})=f((X^{t-n+1},X^{t-n+2},\ldots ,X^{t}),\theta \right)$$

where $X^{t}\in {\mathbb{R}}^{H\times W}$ denotes the traffic congestion at time interval *t*, *f* denotes the prediction model, and *θ* is the learning parameters of this model.

## 4. Methodology

In this section, we first present the architecture of our model, describe the main modules, and give the training algorithm.

### 4.1. AST3DRNet

**Figure 2:**
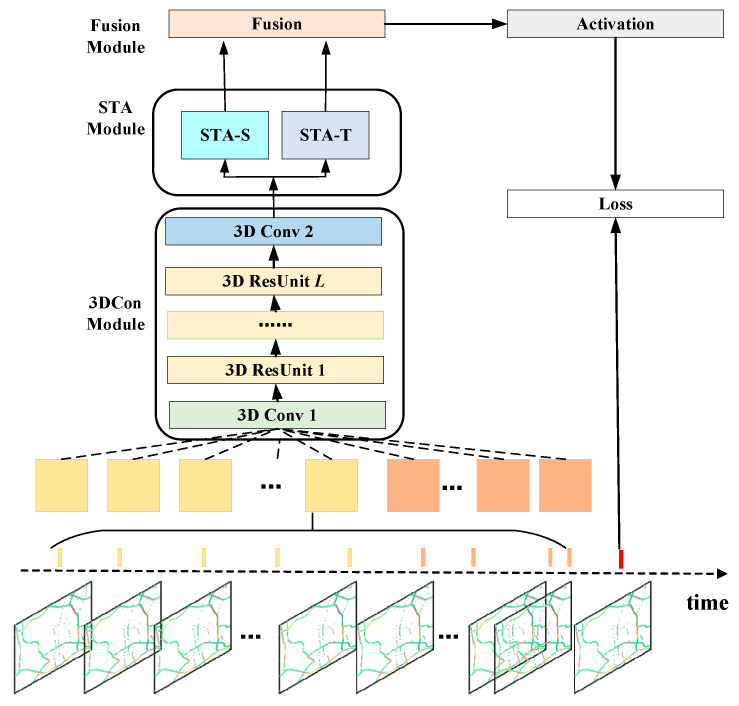
AST3DRNet architecture. 3D Conv: 3D Convolution; 3D ResUnit: 3D Residual Unit; STA-S: Spatial Attention Module; STA-T: Temporal Attention Module.

To solve the problem of modeling the spatial and temporal information of traffic congestion data, we propose an attention-based spatial-temporal 3D residual neural network, named AST3DRNet. Figure 2 shows the architecture of AST3DRNet, which is composed of three major components modeling spatio-temporal correlation and spatio-temporal heterogeneity, respectively.

We first turn a sequence of traffic congestion images captured from the Baidu Map into a sequence of traffic congestion matrices, using the preprocessing process introduced in Section 3. Different traffic congestion matrices represent different congestion levels on road networks at different time intervals. Next, we select congestion matrices representing closeness and period to form the inputs to the network. We then feed these traffic congestion matrices into the 3DCon module to simultaneously model spatio-temporal correlations by stacking 3D residual units. The output of the 3DCon module is fed into the STA module for further modeling of spatio-temporal heterogeneity. In the spatial dimension, the STA module uses the spatial attention sub-module to learn the spatial heterogeneity of spatio-temporal correlations at the element level. In the temporal dimension, the STA module employs the temporal attention sub-module to capture the temporal heterogeneity of spatio-temporal correlations at the matrix level. The outputs of these two sub-modules are fused through the fusion module. Finally, the fusion output is passed through an activation function to generate the predicted values and then the error is calculated with the true values and back-propagated to optimize the network.

### 4.2. The 3DCon Module

**Figure 3:**
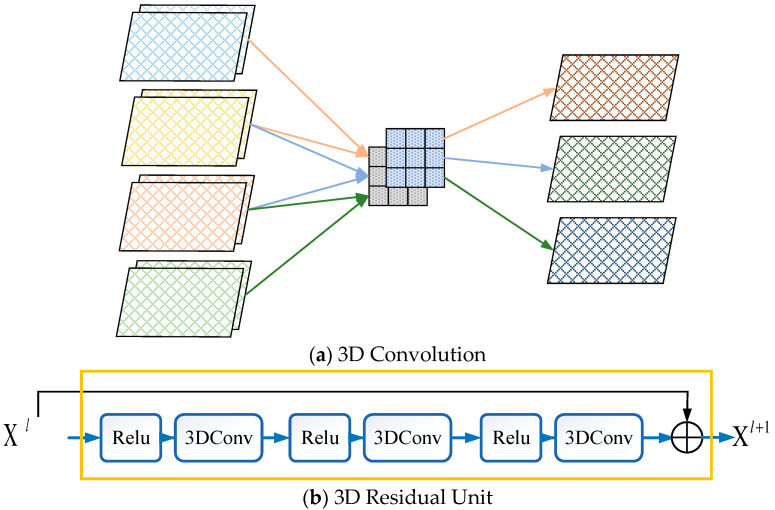
The 3D convolution and 3D residual unit; 3DConv: 3D Convolution.

To model various spatio-temporal correlations of traffic congestion data, we designed a 3DCon module, as shown in Figure 3, which consists of multiple 3D convolutions and 3D residual units.

**3D convolution**. More works [10,41,42] have shown that a stack of 2D convolutions can effectively model nearby spatial dependencies, as well as distant spatial dependencies, in traffic data. However, 2D convolutions lack the ability to capture the temporal dependencies in multiple traffic congestion matrices. After a 2D convolution operation, multiple traffic congestion matrices are compressed into a matrix, and the temporal information among them is missing.

Compared to 2D convolutions, 3D convolutions can simultaneously capture various correlations in both spatial and temporal dimensions. Specifically, when applying a 3D convolution to multiple traffic congestion matrices, the output is also multiple traffic congestion matrices, and the correlation information in the temporal dimension is captured. As shown in Figure 3a, by convolving a 3D filter to four traffic congestion matrices with two channels, the 3D convolution layer generates a feature map consisting of three traffic congestion matrices. On the other hand, one 3D convolution can capture nearby spatial dependencies in the spatial dimension, and a stack of 3D convolutions can gradually model distant spatial dependencies.

A 3D convolution layer can be defined as follows:

(3) $$X^{m}=f(W^{m}*X^{m-1}+b^{m})$$

where $X^{m}\in {\mathbb{R}}^{T\times H\times W}$ denotes the output of this 3D convolution layer, $X^{m-1}\in {\mathbb{R}}^{T\times H\times W}$ denotes the input of the mth 3D convolution layer, * denotes a 3D convolution operation, $W^{m}$ and $b^{m}$ are learnable parameters in this layer, and *f* denotes an activation function. In addition, *T* denotes the number of traffic congestion matrices in the temporal domain, and *H* and *W* refer to the height and width in the spatial domain.

**3D residual unit**. Unfortunately, it is more difficult to train deeper neural networks with more parameters [43]. To address this issue, 2D residual neural networks [43] are proposed to optimize the training process and alleviate the degradation problem.

Inspired by this, 3D residual units are employed in the 3DCon module for better training in AST3DRNet. In our work, we extend the classical 2D residual unit [43] to a 3D residual unit by replacing 2D convolutions with 3D convolutions. The structure of the 3D residual unit is shown in Figure 3b, which is composed of activation functions and 3D convolutions.

The process of calculating a 3D residual unit can be defined as follows:

(4) $$X^{l+1}=X^{l}+F(X^{l};\theta^{l})$$

where $X^{l}\in {\mathbb{R}}^{T\times H\times W}$ denotes the input of the *l*+1th 3D residual unit, *F* is the residual function, and $\theta^{l}$ denotes all learnable parameters in the 3D residual unit.

### 4.3. STA Module

**Figure 4:**
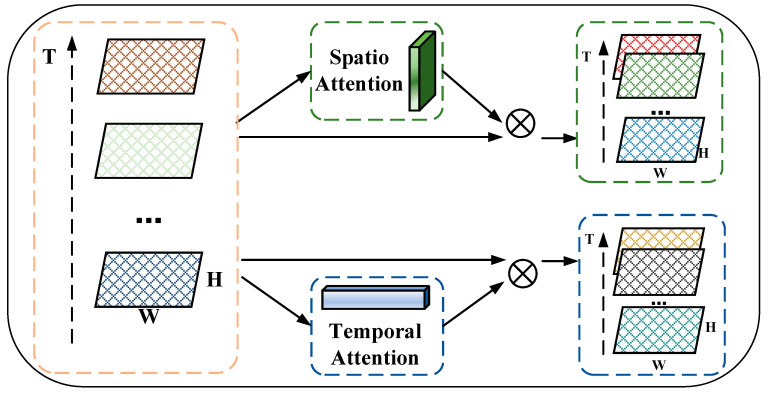
The overview of the STA module.

To model the dynamic spatio-temporal heterogeneity of traffic congestion data, we designed an STA module to emphasize meaningful features and suppress unnecessary ones along the spatial and temporal dimensions. As shown in Figure 4, this spatio-temporal attention module is further divided into two sub-modules, a spatial attention module and a temporal attention module, which can explicitly model different contributions of spatio-temporal correlations in both spatial and temporal dimensions via a self-attention mechanism, respectively.

Given the number of traffic congestion matrices $X_{T}=(X^{t-n+1},X^{t-n+2},\ldots ,X^{t})$ generated by the 3DCon module as input, where a matrix $X^{t-n+i}$ ∈ ℝ*^T^*^×*H*×*W*^, the STA module parallelly infers a 2D spatial attention map *M*_s_ ∈ ℝ*^1^*^×*H*×*W*^ and a 1D temporal attention map *M*_T_ ∈ ℝ*^T^*^×1×1^, respectively. A 2D spatial attention map focuses on the spatial dynamics of spatio-temporal correlations at the element level, while a 1D temporal attention map focuses on the temporal dynamics of spatio-temporal correlations at the matrix level.

The overall spatio-temporal attention process can be defined as

(5) $$X_{T}^{\prime }=M_{s}(X_{T})⊗X_{T}$$

(6) $$X_{T}^{\prime \prime }=M_{T}(X_{T})⊗X_{T}$$

where $M_{s}\in {\mathbb{R}}^{H\times W}$ and $M_{T}\in {\mathbb{R}}^{T}$ denote the spatial attention score and temporal attention score, ⊗ is the element-wise multiplication, $X_{T}^{\prime }\in {\mathbb{R}}^{T\times H*W}$ denotes the spatio-temporal feature map enhanced by a 2D spatial attention map, and $X_{T}^{\prime \prime }\in {\mathbb{R}}^{T\times H*W}$ denotes the spatio-temporal feature map enhanced by a 1D temporal attention map.

It was noted that, during the element-wise multiplication, spatial attention values and temporal attention values are broadcast along the spatial and temporal dimensions, respectively. As a result, in the input, meaningful spatio-temporal features are emphasized and unnecessary ones are suppressed.

**Figure 5:**
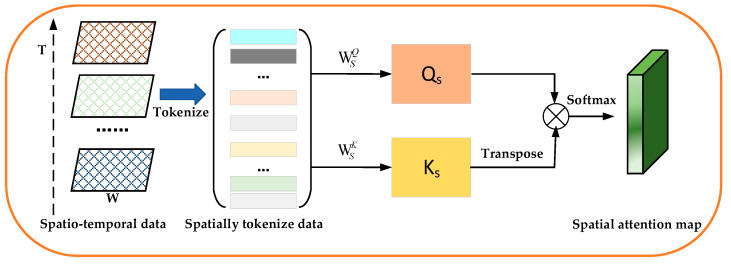
The calculation process of a 2D spatial attention map.

**Spatial attention module**. To compute spatial attention at the element level, we produce a 2D spatial attention map through the self-attention mechanism. In the spatial attention sub-module, we leverage the self-attention mechanism to learn the inter-spatial relationships between elements in a traffic congestion matrix. Figure 5 illustrates the computation process of a 2D spatial attention map.

First, given a sequence of traffic congestion matrices $X_{T}=(X^{t-n+1},X^{t-n+2},\ldots ,X^{t})$, we consider these traffic congestion matrices as a matrix *M* with *T* dimensions. In this matrix, we further consider each element as a word (i.e., token), whose size is (1, *T*). Thus, we reshape the matrix *M* ∈ ℝ*^T^*^×*H*×*W*^ into a sequence of tokens $M=(m_{1},m_{2},\ldots ,m_{H,W})\in {\mathbb{R}}^{H*W\times T}$, where (*H*·*W*) denotes the number of elements and *T* denotes the number of dimensions.

Second, we use the linear projections to compute the query $Q_{S}\in {\mathbb{R}}^{H*W\times T}$ and the key $K_{S}\in {\mathbb{R}}^{H*W\times T}$ for all elements. The operations are defined as

(7) $$Q_{S}=M*W_{S}^{Q}$$

(8) $$K_{S}=M*W_{S}^{k}$$

where the $W_{S}^{Q}\in {\mathbb{R}}^{H*W\times T}$ and $W_{S}^{T}\in {\mathbb{R}}^{H*W\times T}$ are the weight matrices.

Finally, we multiply the attention scores by the input and then add the weighted values together to produce a 2D spatial attention map. In the 2D spatial attention map, the elements with different weights encode which spatial region needs to be emphasized or suppressed for traffic congestion prediction.

The main computation process of a 2D spatial attention map is defined as follows:

(9) $$M_{s}=softmax(Q_{s}{\left(K_{s}\right)}^{T})$$

where $M_{s}\in {\mathbb{R}}^{H\times W}$ denotes the attention map.

**Figure 6:**
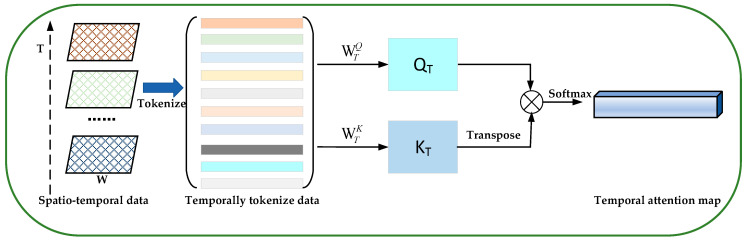
The calculation process of a 1D temporal attention map.

**Temporal attention module**. To compute temporal attention, we generate a 1D temporal attention map through the self-attention mechanism. Different from the spatial attention map, the temporal attention map focuses on matrix-wise attention. In the temporal attention sub-module, we also leverage the self-attention mechanism to learn the inter-temporal relationships between matrices. Figure 6 illustrates the computation process of a 1D temporal attention map.

Given a sequence of traffic congestion matrices $X_{T}=(X^{t-n+1},X^{t-n+2},\ldots ,X^{t})$, we first consider each matrix as a word (i.e., token), whose size is (1, H*W). Each token $e_{i}\in {\mathbb{R}}^{T\times H*W}$ is a matrix, where *T* denotes the number of elements and H*W denotes the number of dimensions.

Similarly, we also use the linear projections to compute the queries $Q_{T}\in {\mathbb{R}}^{H*W\times T}$ and keys $K_{T}\in {\mathbb{R}}^{H*W\times T}$ for all element tokens. The operations are as follows:

(10) $$Q_{T}=M*W_{T}^{Q}$$

(11) $$K_{T}=M*W_{T}^{k}$$

where the $W_{T}^{Q}\in {\mathbb{R}}^{H*W\times T}$ and $W_{T}^{K}\in {\mathbb{R}}^{H*W\times T}$ are the weight matrices. Then, we calculate the attention score through the scaled dot-production operation to produce a 1D temporal attention map. In the 1D temporal attention map, the elements with different weights encode which matrix needs to be emphasized or suppressed for traffic congestion prediction.

It is noted that, to reduce computation overhead, we choose the scaled dot-production operation during the computing of the attention score by dividing by the scaling factor $\sqrt{d_{k}}$.

The main computation process of a 1D temporal attention map is defined as follows:

(12) $$M_{T}=softmax\frac{\left(Q_{T}{\left(K_{T}\right)}^{T}\right)}{\sqrt{d_{k}}}$$

where $M_{T}\in {\mathbb{R}}^{T}$ denotes the 1D temporal attention map, $d_{k}$ denotes the token dimensions.

### 4.4. Fusion Module

To fuse the outputs of the above two sub-modules, we design a fusion module. This can be performed in three steps. First, the outputs of the spatial sub-module and the temporal sub-module are denoted as $X_{T}^{\prime }$ and $X_{T}^{\prime \prime }$, respectively. We fuse $X_{T}^{\prime }$ and $X_{T}^{\prime \prime }$ by assigning different weights to different outputs as follows:

(13) $$X_{f}=W_{T}^{\prime }⊗X_{T}^{\prime }+W_{T}^{\prime \prime }⊗X_{T}^{\prime \prime }$$

where $X_{f}\in {\mathbb{R}}^{T\times H*W}$ denotes the output of the fusion module and $W_{T}^{\prime }$ and $W_{T}^{\prime \prime }$ denote the learnable matrices.

Then, after fusing the two outputs, we aggregate the temporal information $X_{f}$ to generate a matrix with temporal size 1 by applying a 3D convolution operation, formally:

(14) $$\hat{X}=f(W_{f}*X_{f}+b_{f})$$

where $\hat{X}\in {\mathbb{R}}^{H\times W}$ denotes the traffic congestion prediction value and $W_{f}$ and $b_{f}$ are learnable parameters.

Finally, we use the mean square error between the predicted value and the actual value as the loss function, which is defined as

(15) $$W(\theta )=||X-\hat{X}|{|}^{2}$$

where θ denotes all learnable parameters in AST3DRNet, $X\in {\mathbb{R}}^{H\times W}$ denotes the real value, and $\hat{X}\in {\mathbb{R}}^{H\times W}$ denotes the predicted value.

### 4.5. Training Algorithm

Algorithm 1 shows the training process of AST3DRNet. First, we construct the train set. Then, we train our AST3DRNet by minimizing the mean squared error between the predicted congestion level and the real congestion level. The training process is optimized using the Adam optimizer. Finally, the output of this algorithm is a learned AST3DRNet model.

**Algorithm 1.** AST3DRNet
**Input:**
    Historical traffic congestion matrices, {*X*^1^, *X*^2^ …, *X*^*n*^};
**Output:**
    Learned AST3DRNet model;// *construct training instances*1: *M* ← ∅2: **for** *all available time interval t* ∈ {1, 2, …, *n*} **do**3:     XT = (*X*^*t*−*n*+1^, *X*^*t*−*n*+2^ …, *X*^*t*^)//     *X*^*t*+1^ *is the target at time t* + 14:     put a training instance {*X*_*T*_, *X*^*t*+1^} into *M*//*Train the model*5: initialize all learnable parameters *θ* in AST3DRNet6: **repeat**7: select a batch *M*_*b*_ from *M*8: update all the parameters *θ* through minimizing the objective (4) with *M*_*b*_9: **until** stopping criteria are met10: **return** the learned AST3DRNet model.

## 5. Experiment

### 5.1. Dataset

In this research, we focused on traffic congestion conditions in Kunming, China. TrafficKM is a set of citywide traffic congestion maps of the road networks in Kunming, which is captured from Baidu Maps. It was collected from 3 May 2021 to 6 June 2021 (35 days), at a fixed time interval of 5 min. In TrafficKM, the road networks are located within the second ring road in Kunming.

The statistics of TrafficKM are described in Table 1. Traffic congestion maps from 3 May 2021 to 30 May 2021 were used as the training set, and traffic congestion maps from 31 May 2021 to 6 June 2021 were used as the validation set.

### 5.2. Baselines and Metrics

#### 5.2.1. Baselines

To demonstrate the effectiveness of AST3DRNet, we compared it with the following four baseline methods:

(1) HA: The historical average predicts traffic congestion conditions on road networks based on the historical average values in the corresponding period.
(2) CNN: Convolutional neural networks are a popular method for traffic prediction that leverage convolutional filters to capture spatial correlations in traffic data.
(3) ST-ResNet [10]: ST-ResNet is a deep spatio-temporal residual network that employs three residual networks to model spatio-temporal correlations, including temporal closeness, period, trend, and spatially nearby and distant dependencies.
(4) ST-3DNet [15]: ST-3DNet is a deep spatio-temporal 3D convolutional neural network which employs 3D convolutions, residual units, and recalibration blocks to model spatio-temporal correlations and the different effects of spatio-temporal correlations in space.

#### 5.2.2. Evaluation Metrics

To evaluate the prediction performance of our model, we choose three evaluation metrics, i.e., Root Mean Square Error (RMSE), Mean Absolute Error (MAE), and Mean Absolute Percentage Error (MAPE). For all these metrics, smaller values indicate better prediction performance. Equations (16)–(18) define RMSE, MAE, and MAPE, where $X\in {\mathbb{R}}^{H,W}$ denotes the real value and $\hat{X}\in {\mathbb{R}}^{H,W}$ denotes the predicted value, respectively.

(16) $$RMSE=\sqrt{\frac{1}{n}\sum_{i=1}^{n}{\left(X-\hat{X}\right)}^{2}}$$

(17) $$MAE=\frac{1}{n}\sum_{i=1}^{n}\left|X-\left.\hat{X}\right|\right.$$

(18) $$MAPE=\sum_{i=1}^{n}\left|\left.\frac{X-\hat{X}}{X}\right|\right.$$

### 5.3. Experiment Settings

#### 5.3.1. Training Settings

We implement our AST3DRNet as well as baselines using Pytorch on an NVIDIA RTX A6000 GPU with 64 GB RAM and 48 GB GPU memory. These models were trained on the TrafficKM dataset, where we chose the last 7 days (20%) as testing data and used the remaining (80%) as training data. The Min-Max normalization method was used to normalize the congestion level to [0, 1] and we compared the ground truth with the re-scale of the predicted value. In the training process, the learning rate was set to 5 × 10^−7^, the batch size was set to 4, the training epoch was set to 300, and the activation function was ReLU. In addition, the lengths of the closeness and period were set to 7 and 3, respectively.

#### 5.3.2. Model Settings

The details of our proposed model are listed in Table 2. The model has seven layers, including 12 3D convolutions. The input of the model is a sequence of traffic congestion maps with sizes (10, 224, 224, 1), where the first number denotes a sequence of 10 traffic congestion maps, and the second and third number denote the row and column of a traffic congestion map. The input layer is followed by the 3D convolution layer (layer 1) with 64 3D filters of size (3 × 3 × 3) and strides of (1, 1, 1). This layer 1 is followed by the 3DCon module, which consists of layer 2, layer 3, and layer 4. These layers, 2, 3, and 4, all have a stack of 3 3D convolutions, which encode the input (10, 224, 224, 64) to (10, 224, 224, 1024). Layer 5 further converts (10, 224, 224, 1024) to (10, 224, 224, 1) and feeds it to the STA module by a 3D convolution with 1 3D filter of size (1 × 1 × 1). The STA module consists of a spatial attention sub-module and a temporal attention sub-module, which learns spatio-temporal heterogeneity from the input (10, 224, 224, 1) through the self-attention mechanism. The fusion module (layer 7) fuses the outputs of the two sub-modules. The output of the model is a prediction matrix of size (1, 224, 224, 1).

### 5.4. Results and Analysis

#### 5.4.1. Comparison with the Baseline Methods

**Figure 7:**
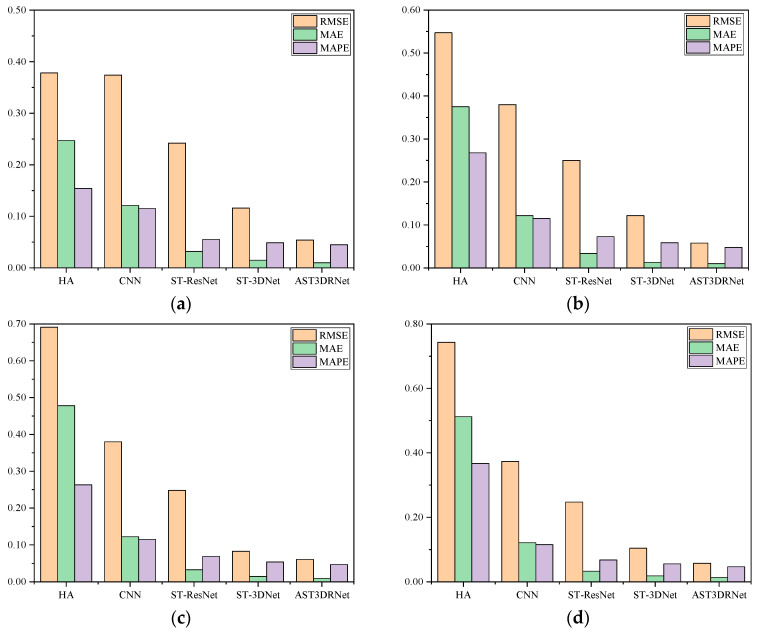
Comparison of AST3DRNet and other four baselines for peak and off-peak periods. (**a**) Comparison of AST3DRNet and other four baseline methods during an off-peak period of 00:00~03:00. (**b**) Comparison of AST3DRNet and other four baseline methods during a peak period of 07:00~10:00. (**c**) Comparison of AST3DRNet and other four baseline methods during a peak period of 11:00~14:00. (**d**) Comparison of AST3DRNet and other four baseline methods during a peak period of 17:00~20:00.

(1) In this experiment, we compare AST3DRNet with four baselines on short-term (5/10/15 min) traffic congestion prediction. Table 3 shows that our proposed model outperforms all the other models. More specifically, our AST3DRNet has a relatively lower 59.05% RMSE, 64.69% lower MAE, and 48.22% lower MAPE on average than HA, CNN, ST-ResNet, and ST-3DRNet. Generally, as the time span for prediction increases, the prediction performance of all five models drops. However, HA’s performance drops dramatically. The reason is that HA is unable to model the non-linear correlations of traffic data and is sensitive to non-smoothed traffic data. Moreover, CNN is worse than ST-ResNet, and ST-3DRNet. This is because CNN only captures the spatial correlations and fails to model the temporal correlations. Compared with ST-ResNet and ST-3DRNet, our proposed model has a better result. This indicates that AST3DRNet not only captures simultaneously spatial and temporal correlations using 3D convolutions but also models spatial and temporal heterogeneity using two attentional sub-modules.

(2) In this experiment, we compared AST3DRNet with four baselines on 5 min traffic congestion prediction in the daytime (08:00–20:00), as shown in Table 4. It can be observed that AST3DRNet has better prediction performance for all metrics, i.e., RMSE, MAE, and MAPE. More specifically, AST3DRNet has a relatively lower 87.42% RMSE, 83.45% lower MAE, and 85.10% lower MAPE than HA. Then, we compared AST3DRNet against CNN and ST-ResNet. On average, AST3DRNet has a relatively lower RMSE of 73.95%, a 76.44% lower MAE, and a 34.82% lower MAPE than them. Finally, compared with ST-3DNet, AST3DRNet has a relatively lower 13.95% RMSE, 33.43% lower MAE, and 4.92% lower MAPE. This indicates that our model performs well in non-smoothed traffic data during the daytime.

(3) In this experiment, we compared AST3DRNet against four baseline methods for peak and off-peak periods. Figure 7 shows that our model consistently has a better performance in four different periods. More specifically, for the off-peak period of 00:00~03:00, AST3DRNet always performs better than HA, CNN, and ST-ResNet, and has slight advantages over ST-3DNet. For three peak periods, AST3DRNet always has relatively lower RMSE, MAE, and MAPE than these four baselines. This indicates that our model performs well in different periods.

#### 5.4.2. Ablation Analysis

In this experiment, we verified the effectiveness of the STA module. For this ablation study, we chose three model variants, namely ST-3DRNet, 3DRNet-SA, and ST-3DRNet-TA. ST-3DRNet includes 3DCon and a fusion module without the spatio-temporal attention module. ST-3DRNet-SA consists of the 3DCon module, the fusion module, and the spatial attention sub-module without the temporal attention sub-module. ST-3DRNet-SA is composed of the 3DCon module, the fusion module, and the temporal attention sub-module without the spatial attention sub-module.

**Figure 8:**
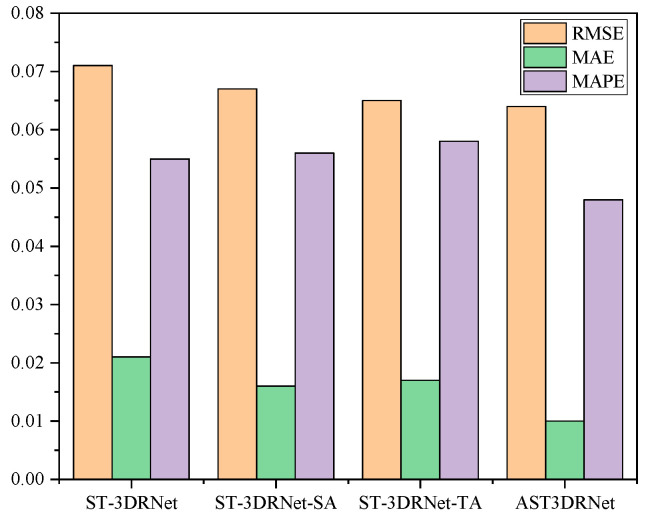
Comparison of AST3DRNet and three model variants.

Figure 8 shows the comparison of AST3DRNet and three different model variants. It can be seen that our AST3DRNet has a better performance for all metrics than the three model variants. Specifically, ST-3DRNet performs worse than all other models. This is because it cannot model spatio-temporal heterogeneity. Further, ST-3DRNet-SA and ST-3DRNet-TA perform better than ST-3DRNet because they can capture spatial heterogeneity and temporal heterogeneity, respectively. Finally, our AST3DRNet reduces the RMSE to 0.072, the MAE to 0.010, and the MAPE to 0.048, which demonstrates the superior ability in the feature representation of spatio-temporal heterogeneity.

#### 5.4.3. Visualization

**Figure 9:**
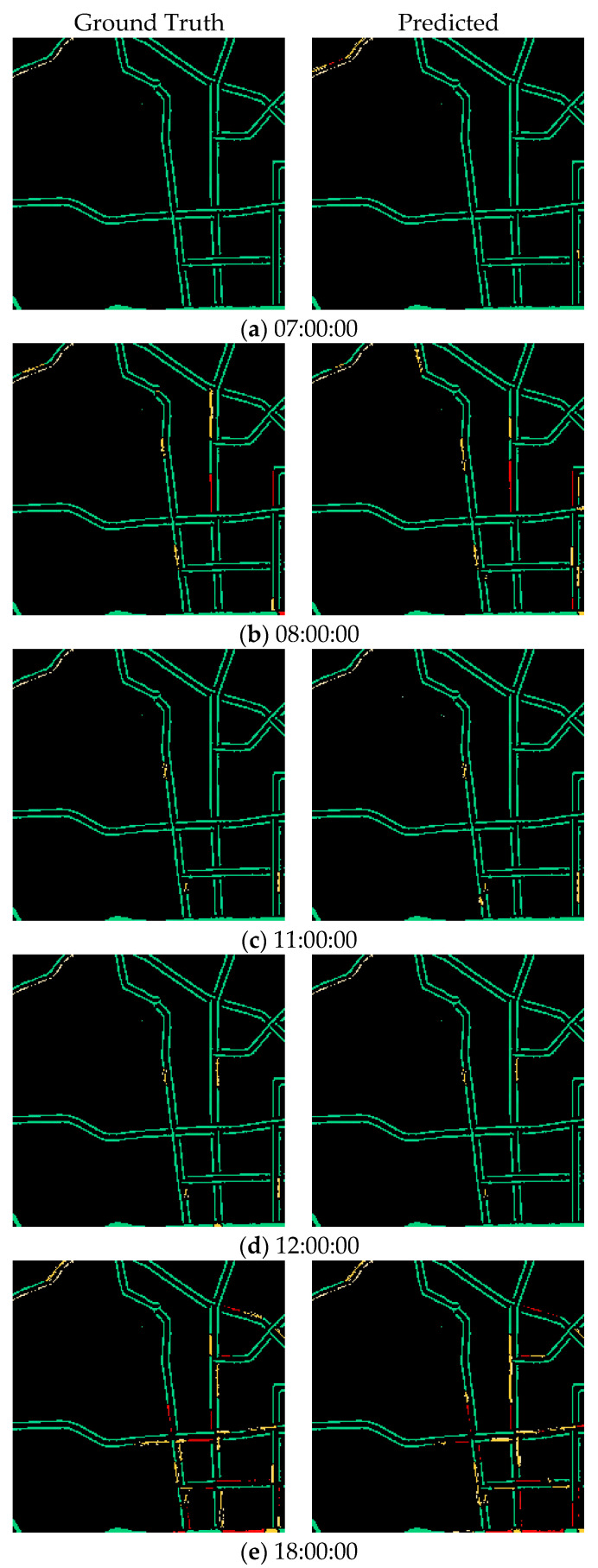

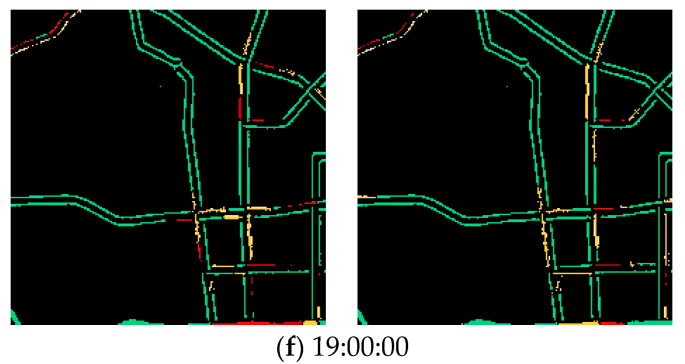
The visualization prediction results of AST3DRNet.

In this experiment, the results show that AST3DRNet performs well for different periods. Specifically, Table 5 shows the traffic congestion prediction accuracy on 4 June 2021 from 00:70 to 24:00 at every hour, where 00:00~03:00 indicates the off-peak period and 07:00~10:00, 11:00~14:00, and 17:00~20:00 indicate the three peak periods, respectively. It can be observed that the RMSE, MAPE, and MAE of the off-peak period are always lower than those of the three peak periods. This is because, with the increasing number of vehicles on road networks during the peak periods, the variability of traffic congestion conditions becomes more dramatic. In addition, Figure 9 shows the visualization prediction results of AST3DRNet, which compares the ground truth with its corresponding prediction values on 4 June 2021. It can be observed that the error color between the real congestion level and the predicted congestion level is always small. This indicates that our AST3DRNet can achieve better prediction accuracy.

## 6. Conclusions

In this paper, we propose an attention based spatio-temporal 3D residual neural network (AST3DRNet) for traffic congestion prediction, which can learn spatio-temporal representation from traffic data. We innovatively introduce a self-attention mechanism into the traffic congestion prediction domain. We propose a 3DCon module to capture various spatio-temporal correlations and an STA module to model dynamic spatio-temporal heterogeneity. Our proposed module outperforms four baseline methods on a real-world traffic congestion dataset and obtains a better prediction performance.

It is worth noting that AST3DRNet is a general prediction model, which is suitable for other traffic prediction tasks, such as traffic flow prediction and traffic speed prediction.

In future work, we will apply a pure transformer to the traffic prediction domain without CNNs, RNNs, or GCNs. How to adaptively apply a standard transformer to forecast traffic conditions with the fewest modifications will be addressed in our future work.

## Figures and Tables

**Table 1:** Statistics of the traffic congestion dataset.

Dataset	TrafficKM
location	In Kunming
time span	3 May 2021–6 June 2021
time interval	5 min
raster size	(224, 224)
area	6.584 km^2^
number of available time intervals	10,080

**Table 2:** Hyperparameters of the model.

Layer	Name	Parameters	Dimensions
Input	-	-	(10, 224, 224, 1)
Layer 1	3DCon	64, 3 × 3 × 3	(10, 224, 224, 64)
Layer 2		64, 1 × 1 × 1	(10, 224, 224, 64)
		64, 3 × 3 × 3	
		256, 1 × 1 × 1	(10, 224, 224, 256)
Layer 3		128, 1 × 1 × 1	(10, 224, 224, 128)
		128, 3 × 3 × 3	
		512, 1 × 1 × 1	(10, 224, 224, 512)
Layer 4		256, 1 × 1 × 1	(10, 224, 224, 256)
		256, 3 × 3 × 3	
		1024, 1 × 1 × 1	(10, 224, 224, 1024)
Layer 5		1, 1 × 1 × 1	(10, 224, 224, 1)
Layer 6	AST	1, 1 × 1	(1, 224, 224)
		1, 1 × 1	(1, 224, 224)
		1, 1 × 1	(1, 224, 224)
		1, 1 × 1	(1, 224, 224)
Layer 7	Fusion	1, 1 × 1 × 1	(1, 224, 224)
Output	-	-	(1, 224, 224, 1)

**Table 3:** Comparison of AST3DRNet with four baselines on the short-term traffic congestion prediction. The best result is marked in bold.

Baseline	5 min			10 min			15 min		
	RMSE	MAE	MAPE	RMSE	MAE	MAPE	RMSE	MAE	MAPE
HA	0.488	0.252	0.272	0.537	0.344	0.338	0.671	0.427	0.414
CNN	0.233	0.093	0.103	0.298	0.141	0.183	0.394	0.209	0.275
ST-ResNet	0.197	0.024	0.062	0.227	0.039	0.087	0.305	0.047	0.098
ST-3DRNet	0.081	0.015	0.051	0.092	0.021	0.067	0.113	0.035	0.083
**AST3DRNet**	**0.072**	**0.01**	**0.048**	**0.083**	**0.019**	**0.053**	**0.093**	**0.025**	**0.071**

**Table 4:** Prediction performance of AST3DRNet and other four baseline methods for 08:00 to 20:00 on 31 May 2021. The best result is marked in bold.

Date and Time	RMSE					MAE					MAPE				
	HA	CNN	ST-ResNet	ST-3DNet	AST3DRNet	HA	CNN	ST-ResNet	ST-3DNet	AST3DRNet	HA	CNN	ST-ResNet	ST-3DNet	AST3DRNet
5-03 08:00	0.556	0.393	0.261	0.121	**0.078**	0.389	0.135	0.052	0.021	**0.012**	0.276	0.122	0.081	0.063	**0.052**
5-03 09:00	0.543	0.372	0.257	0.129	**0.075**	0.376	0.129	0.049	0.018	**0.012**	0.268	0.117	0.078	0.057	**0.050**
5-03 10:00	0.549	0.379	0.259	0.128	**0.076**	0.381	0.131	0.050	0.018	**0.012**	0.271	0.119	0.079	0.056	**0.051**
5-03 11:00	0.683	0.388	0.247	0.089	**0.075**	0.469	0.135	0.046	0.014	**0.009**	0.297	0.121	0.076	0.058	**0.050**
5-03 12:00	0.689	0.387	0.249	0.097	**0.076**	0.471	0.134	0.047	0.015	**0.011**	0.299	0.121	0.078	0.059	**0.051**
5-03 13:00	0.709	0.392	0.254	0.114	**0.074**	0.493	0.140	0.049	0.016	**0.010**	0.309	0.129	0.079	0.057	**0.049**
5-03 14:00	0.712	0.399	0.251	0.105	**0.071**	0.487	0.141	0.049	0.015	**0.010**	0.310	0.129	0.078	0.057	**0.048**
5-03 15:00	0.612	0.379	0.257	0.116	**0.077**	0.411	0.128	0.050	0.014	**0.010**	0.281	0.118	0.078	0.056	**0.049**
5-03 16:00	0.645	0.380	0.263	0.115	**0.076**	0.427	0.131	0.058	0.014	**0.009**	0.293	0.120	0.087	0.057	**0.049**
5-03 17:00	0.792	0.411	0.253	0.103	**0.070**	0.519	0.149	0.048	0.013	**0.011**	0.401	0.131	0.080	0.057	**0.047**
5-03 18:00	0.813	0.408	0.249	0.107	**0.073**	0.526	0.148	0.047	0.014	**0.010**	0.409	0.130	0.079	0.056	**0.048**
5-03 19:00	0.822	0.395	0.250	0.101	**0.071**	0.534	0.147	0.047	0.013	**0.010**	0.412	0.129	0.080	0.056	**0.048**
5-03 20:00	0.842	0.413	0.248	0.099	**0.070**	0.541	0.150	0.047	0.013	**0.009**	0.419	0.131	0.079	0.055	**0.049**

**Table 5:** Traffic congestion prediction accuracy on 4 June 2021.

Time	RMSE	MAE	MAPE
00:00~03:00	0.044	0.009	0.037
07:00~10:00	0.058	0.010	0.048
11:00~14:00	0.061	0.011	0.047
17:00~20:00	0.058	0.010	0.047

## Data Availability

Data is contained within the article.
